# Moving Just Like You: Motor Interference Depends on Similar Motility of Agent and Observer

**DOI:** 10.1371/journal.pone.0039637

**Published:** 2012-06-27

**Authors:** Aleksandra Kupferberg, Markus Huber, Bartosz Helfer, Claus Lenz, Alois Knoll, Stefan Glasauer

**Affiliations:** 1 Center for Sensorimotor Research, Institute of Clinical Neurosciences, Ludwig-Maximilian University Munich, Germany; 2 Graduate School for Systemic Neurosciences GSN, Ludwig-Maximilian University Munich, Germany; 3 Robotics and Embedded Systems, Technical University Munich, Germany; Katholieke Universiteit Leuven, Belgium

## Abstract

Recent findings in neuroscience suggest an overlap between brain regions involved in the execution of movement and perception of another’s movement. This so-called “action-perception coupling” is supposed to serve our ability to automatically infer the goals and intentions of others by internal simulation of their actions. A consequence of this coupling is motor interference (MI), the effect of movement observation on the trajectory of one’s own movement. Previous studies emphasized that various features of the observed agent determine the degree of MI, but could not clarify how human-like an agent has to be for its movements to elicit MI and, more importantly, what ‘human-like’ means in the context of MI. Thus, we investigated in several experiments how different aspects of appearance and motility of the observed agent influence motor interference (MI). Participants performed arm movements in horizontal and vertical directions while observing videos of a human, a humanoid robot, or an industrial robot arm with either artificial (industrial) or human-like joint configurations. Our results show that, given a human-like joint configuration, MI was elicited by observing arm movements of both humanoid and industrial robots. However, if the joint configuration of the robot did not resemble that of the human arm, MI could longer be demonstrated. Our findings present evidence for the importance of human-like joint configuration rather than other human-like features for perception-action coupling when observing inanimate agents.

## Introduction

Engaging in interactions with other individuals requires anticipating their behaviors, sharing representations and coordinating actions with them [1]. The capacity to understand goals and intentions emerges early and universally in humans and is a basic precondition for the interpretation and prediction of others’ actions, be it other humans, animals, or inanimate agents. But what is the reason for easiness and intuitiveness of action understanding? The common coding theory states that perception of an action leads to simulative production of that action on the part of the observer [2], [3]. The neural basis for this so called "action-perception coupling" hypothesis has come with the discovery of the mirror neurons in the premotor cortex of macaques, which are activated both when a monkey performs a specific action and when it passively observes the experimenter perform that same action [4], [5]. It has been argued, that in humans, the mirror neuron system (MNS) facilitates action understanding, based on the suggestion that neural simulation of observed actions allows us to plan our own actions and also to interpret the actions of others using our own previous experience while performing these actions (simulation theory) [6], [7], [8].

If a part of the central motor systems becomes activated during the observation of action, what happens when we attempt to make an action while observing a qualitatively different (incongruent) action? In this case, the motor program (or representation) associated with the observed movement interferes with the outgoing motor output for the intended movement. Thus, caused by the internal neuronal simulation during action observation, the perception of an action leads to simulative production of that action on the part of the observer, facilitating a similar action (motor resonance) and interfering with a different action (motor interference) [2], [3], [9]. While motor resonance becomes obvious in mimicking actions of our interaction partners, motor interference (MI) can be observed as an increase of variance in our own movement trajectory while watching an incompatible movement either face-to-face or in video [10].

However, it is not clear whether motor resonance and thus MI need a tight match between one’s own and the observed agent’s physical features to emerge. These features could be, for example, presence of a body, head, face, extremities, natural movement kinematics or capability of self-propulsion. Previous studies indicated that it is not sufficient that the overall pattern of the observed movement matches that of the observer (e.g., moving an arm from side to side), but that a biological [11], [12] or at least a quasi-biological [13] movement profile is required to trigger MI. None of these previous studies was able to disentangle whether biological motion is the only requirement for MI or whether other morphological similarities between agent and observer have to be present. A recent study investigating motor coordination proposed that rather than any single feature the overall perception of the agent as a “social entity”, e.g., elicited by top-down information, is the critical factor [14].

In the absence of top-down cues, the question remains which basic features of the observed agent and the observer have to match for MI to occur. In the present study we investigated what aspects in the appearance (for example, head and body), motility (ability to move resulting from the joint configuration) and movement kinematics (variability, velocity) of the observed agent are responsible for triggering MI during observation of incongruent movements. If quasi-biological motion was sufficient, we expected to see an effect of MI on movement production while viewing videos of incongruent movements performed by an industrial robot arm. Alternatively, absence of MI during observation of quasi-biological motion of an industrial robot arm might be caused by its artificial motility, which results from the joint configuration that does not match the one of the human arm. To test this possibility, we presented subjects with the rotated video of the industrial robot arm. This rotated configuration of the arm was equal to the arm of a humanoid robot shown in our previous study to trigger MI [13], except that its appearance was still that of an industrial robot. As in Kupferberg et al. [13], we used the MI paradigm described previously [10] but replaced live presentations with video clips.

## Materials and Methods

### Subjects

Twelve female and ten male right-handed graduate students from the local Department of Neurology were tested in the present experiments (age range: 20–25 years). In the previous experiment [13] performed with the humanoid robot JAST, ten female and fifteen male right-handed graduate students have participated (age range: 26–35 years). The experiments were approved by the ethics committee of the medical faculty of the LMU, conducted in accordance with the Declaration of Helsinki, and all participants gave their written informed consent.

### Stimuli

**Figure 1:**
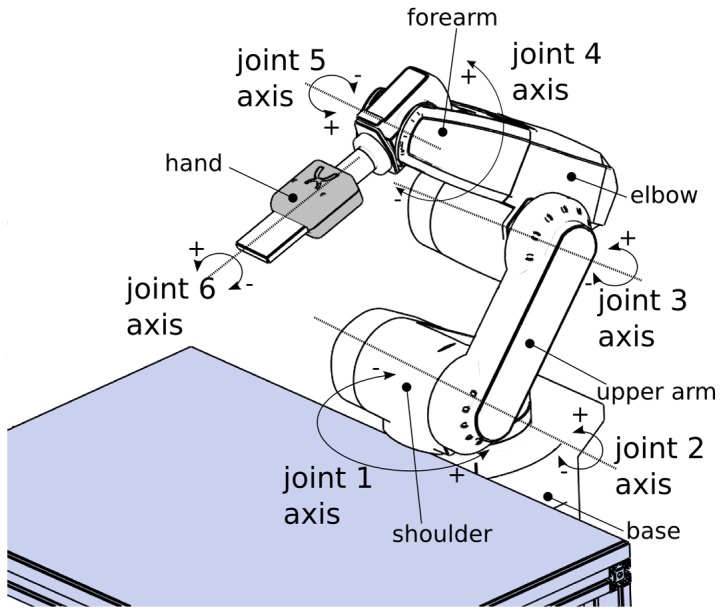
Drawing of the robot arm. The robot arm JAHIR, which was used in the experiment, consisted of a base, upper arm and forearm connected though joints.

**Figure 2:**
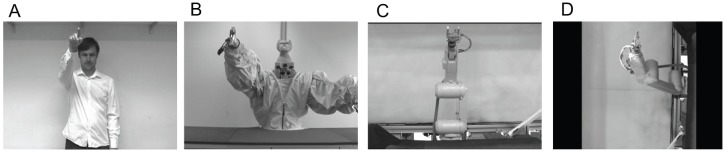
Video Screenshots. Screenshots from the videos of the different agents presented to the subjects in the previous [13] and the current experiment. The participants were instructed to perform horizontal or vertical movements while viewing the videos and fixating on the right hand of A) a human agent (MH), B) the humanoid robot JAST [13], C) the industrial robot arm JAHIR and D) JAHIR rotated, which performed congruent or incongruent movements. The experimenter shown in A has given written informed consent (as outlined in the PLoS consent form) to publication of his photograph.

The videos of the human agent and the industrial robot arm JAHIR (Mitsubishi, RV-6SL; Fig. 1; [15] were rear-projected in a pseudo-randomized order on a white screen (120 cm×160 cm) located ca. 1.5 m in front of the participant. The use of video presentations allowed us to control for the between-trial variability in the movements of the human agent, which otherwise might have been an additional factor causing increased variability in the subjects' movements. The human agent shown in the videos was always the same person (MH, see Fig. 2a).

In contrast to the humanoid robot JAST used in the previous experiment [13], which had an “animal” head and two industrial arms covered with a plastic “shirt”, the robot arm JAHIR consisted of one of the arms of JAST and has been left uncovered. Thus, both robots had arms with six degrees of freedom and were capable of producing movements with a minimum-jerk (quasi-biological) velocity profile [16]. The forearm ended in a metallic gripper connected by a wrist joint (s. Fig. 2c).

JAHIR consisted of a base, an upper arm and a forearm which are connected through a shoulder joint and an elbow joint (shown by circle arrows in the Fig. 1) and was mounted on a working bench (Fig. 2c). To make the joint configuration resemble the joint configuration of the human arm for additional testing, for the second test condition, the video of JAHIR was rotated 90° to the left (s. Fig 2d). Thus, the configuration corresponded to that of JAST.

During the vertical condition, JAHIR performed an up-and-down movement with the amplitude of 50 cm using its shoulder joint (J2 axis) and the elbow joint (J3 axis) (s. Fig. 2c). During the horizontal condition the movement was performed by the shoulder (J1 axis and J2 axis), the elbow (J3 axis), and the wrist joint (J5 axis). By implementing minimum-jerk profiles [15] we achieved a quasi-biological acceleration and deceleration of each movement resulting in a bell-shaped velocity profile, where mathematically the derivative of acceleration (jerk) is minimized over the movement. Thus, by preventing abrupt changes in movement velocity, in contrast to a constant velocity profile, minimum-jerk movements look smoother and more natural [17].

The human experimenter depicted in the video clip also performed horizontal and vertical movements with the amplitude of 50 cm. To make robot gripper and human hand more similar, the hand had been painted in silver colour (s. Fig. 2 a).

### Procedure

**Figure 3:**
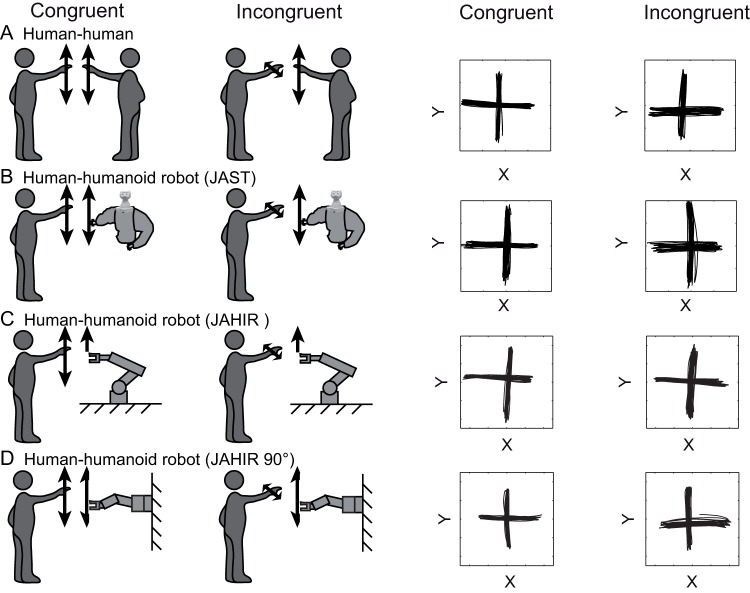
Overview of all experimental conditions. Summary of all conditions of the present (A, C, D) and the previous experiments (A, B) [13]. Left: experimental conditions (only vertical agent movement is shown). Right: examples of movement trajectories performed by the observing subjects.

In the previous [13] and present experiments the subjects were instructed to perform ca. 50-cm amplitude horizontal (H) or vertical (V) rhythmic right arm movements directed by the shoulder joint while watching the hand of the human experimenter or the robot gripper respectively. In an additional baseline control condition, the subjects were instructed to produce horizontal and vertical movements without looking at their arm. The observed agent (H, human or R, robotic) performed either spatially congruent (C, same direction) or incongruent (I, perpendicular) movements (frequency: 0.5 Hz) with the right arm. Like in the previous experiment, this resulted in a 2×2×2 experiment design with eight experimental conditions and three factors: (1) movement PLANE (Horizontal/Vertical), (2) CONGRUENCY (Congruent/Incongruent), and (3) observed AGENT (Human/Robot) plus 2 baselines. In the additional experiment, 10 participants were retested while viewing horizontal and vertical, congruent and incongruent videos of the robot JAHIR which was rotated 90 degrees to the right and scaled in a way that the movements of the robot arm had the same horizontal and vertical amplitude in both directions as in the original video. For an overview of all conditions, see Fig. 3.

One trial (duration: ca. 30 s) was performed for each condition. At the start of each new condition, the participants were informed (by an instruction appearing on the screen) of the plane in which to move their arm and instructed to keep in phase with the agent’s movements.

### Data acquisition and analysis

The kinematics of the endpoint of the right index finger of each participant was recorded at 240 Hz using the magnetic-field based motion tracking system Polhemus Liberty by fixating a small 1×1 cm sensor on the finger tip. After data acquisition, fingertip positions of subjects were filtered with a 20-Hz second order Butterworth filter. The data from each trial was split into single movement segments (from right to left and from top to the bottom and vice versa) by finding data points at which the x- and y-values reached their maxima and minima.

As a standard measure of MI, most previous studies used variance or standard deviation (SD) of fingertip position of the observer from the *instructed* axis of movement ([10], [18], [19], [20], [21], [22]; for an exception see [11], [12]. This standard measure of fingertip SD relies on a spatial frame of reference, i.e., the instructed horizontal or vertical direction of movement, but is composed of several components contributing to the overall variability and thus to the quantification of MI: 1) tilt away from the instructed direction, 2) variability of movement direction within a single trial, and 3) curvature of the individual movements. Evidently, reliance on a spatial reference frame to measure MI might induce higher SD if the movement of the observed agent deviates from the instructed direction and thus make comparisons between experiments more difficult. However, so far, no study has examined the contribution of each component to MI. Therefore, we also investigated the components contributing to the quantification of MI.

**Figure 4:**
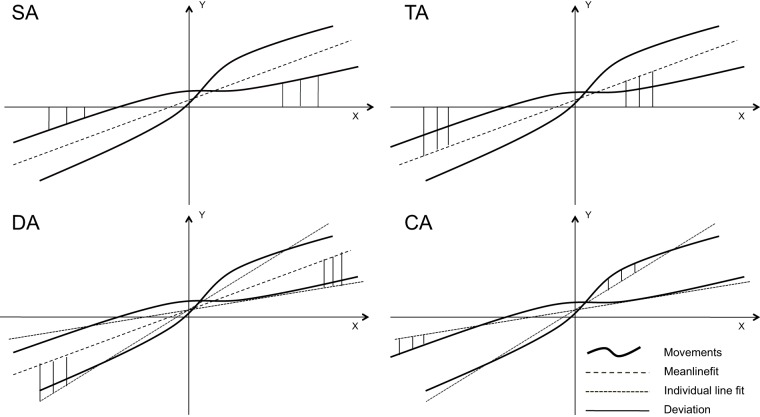
Illustration of the types of analysis. Four types of analyses performed on the present experiment: standard analysis (SA), tilt analysis (TA), deviation analysis (DA), curvature analysis (CA). In the SA, we calculated the deviations of the individual movement from the horizontal or vertical axis. In the TA, we calculated the tilt (or shift) of the overall line fit from the horizontal or vertical axis. In the DA, the deviations of line fits for individual movements from the overall line fit have been calculated. Finally, in CA, we calculated the deviations of every single movement from the straight line fitting this movement.

The standard deviation of fingertip position from the y-axis in case of instructed vertical movement and x-axis in case of instructed horizontal movement was calculated for each subject and movement (see Fig. 4, SA). The average standard deviation for each condition and subject was used for statistical analysis. This analysis will further be referred to as *standard analysis* (*SA*).

To investigate the different types of contributions to SA we applied 3 additional types of analyses to the data. To determine the amount of curvature of each individual movement in a 30 s trial, a least-squares individual line fit was determined for *each* movement and the standard deviation (see Fig. 4, dotted lines in CA) of the actual movement from this line was calculated. The average of SD across all single movements was calculated for each trial to estimate the curvature. This kind of analysis will further be referred as *curvature analysis (CA)*. A similar analysis has been used in two previous studies investigating MI [11], [12].

In the second analysis method we determined the best line fit to *all* individual movements in a 30 s trial (s. Fig 4, dashed lines in DA) and then calculated the standard deviation of each *individual* line fit from this *overall line fit* (see Fig. 4 DA). This overall line fit, which represents the average direction of movement, does not necessarily need to correspond to the instructed movement along the horizontal or vertical axis (like assumed in the *standard analysis*) but might be tilted or shifted with respect to it. Thus, the deviations in this type of analysis (*deviation analysis, DA*) are composed of the shift (or tilt) of every single movement with respect to the overall plane of movement.

In the final analysis, we determined the deviation of the average direction of movement (overall line fit) from the x-axis (see Fig. 4, TA) in case of horizontal movement and the y-axis in case of vertical movement. This type of analysis will be referred to as *tilt analysis* (*TA*).

To test if there is a correlation between these different contributing factors and the standard analysis (SA) we closer investigated the observation of a human agent, since the database for these cases was the largest. First, we excluded outliers from the SA data of each condition until none of the values fell out of the 95% interval. Due to this outlier rejection, 19 data points (4.6%) were excluded from the analysis. For the complete statistical analysis (all factors), this resulted in excluding 5 subjects from the previous experiment [13] and 7 subjects from the present experiment. For the standard analysis, pooling across movement direction (see Results for justification) after outlier removal allowed us to use data from all but two subjects (one from each experiment). For two other subjects from the present experiment, due to technical difficulties, data could be obtained only for observation of the robot but not observation of the human agent.

The correlation analysis was performed across values obtained by different types of analysis (SA, CA, DA, TA) for the four conditions of human agent observation: horizontal congruent (HC), horizontal incongruent (HI), vertical congruent (VC) and vertical incongruent (VI). We detected a correlation (from moderate till strong) between each of the contributing factors CA, DA, TA and SA in most of tested conditions: HC, HI, VC and VI. Therefore, to show that values from the DA, CA and TA are contributors of the SA, we performed a multiple linear regression with these factors as independent variables and SA values as dependent variables in the following conditions: HC, HI, VC and VI.

## Results

### Standard analysis

**Figure 5:**
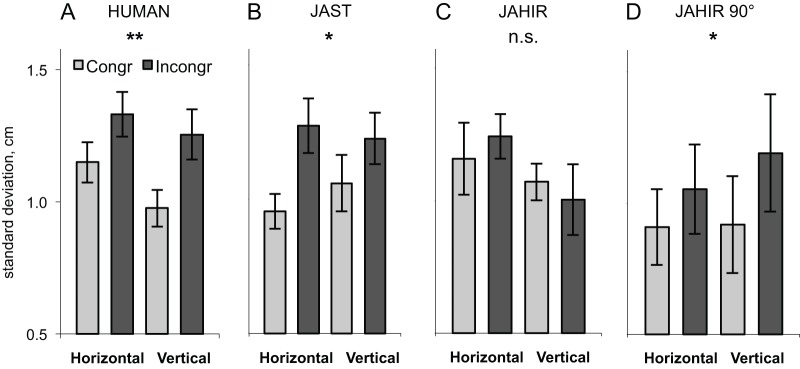
Results using the standard analysis. Standard deviation (SD) of movement from the instructed movement plane during observation of incongruent and congruent movements of the human agent (A), humanoid robot JAST (B), industrial robot JAHIR (C) and rotated industrial robot JAHIR90° (D). Data from all subjects (including [13]), i.e., each graph represents a different number of subjects (see text). Error bars represent standard error of the mean. Stars denote significance (** p<0.01; * p<0.05).

To compare the MI effect elicited by action observation of the human, the humanoid robot (JAST [13]), and the industrial robot (JAHIR, artificial joint configuration; present experiment) we used the standard analysis. We combined all data to yield a repeated-measures ANOVA design with 3 within-subjects factors and one between-subjects factor. We used MOVEMENT PLANE (horizontal/vertical), CONGRUENCY (congruent/incongruent), and AGENT (human/robot) as within-subject factors and ROBOT (humanoid JAST/industrial JAHIR) as between-subjects factor resulting in a mixed 2*2*2 within-subject-design with 2 between-subject conditions. The combined analysis (33 subjects; conditions a, b, and c in Figs. 2 and 3) revealed a significant main effect for CONGRUENCY [F(1,31) = 10.5; p<0.0028] that confirmed motor interference. The strength of motor interference depended on whether the agent was a human or a robot and the type of the robot as shown by a significant three-way interaction AGENT×CONGRUENCY×ROBOT [F(1,31) = 4.38; p = 0.044] (Fig. 5).

Since MOVEMENT PLANE became neither significant as main effect nor as interaction, we pooled data across this factor. This allowed us to include data from subjects who previously were excluded due to an outlier (see Methods). The pooled analysis (43 subjects) with CONGRUENCY (congruent/incongruent), and AGENT (human/robot) as within-subject factors and ROBOT (humanoid JAST/industrial JAHIR) as between-subjects factor resulted in a main effect for CONGRUENCY [F(1,41) = 20.2; p<0.0001] and a significant three-way interaction AGENT×CONGRUENCY×ROBOT [F(1,41) = 4.53; p = 0.039], confirming the results above.

To further investigate how subjects reacted to the observation of human, humanoid robot, industrial robot and rotated industrial robot (industrial 90°) movement, we performed separate post hoc analyses (repeated measures ANOVA) with CONGRUENCY (congruent/incongruent) as within-subject factor. This analysis revealed an effect of congruency for the human agent [F(1,42) = 18.5; p<0.0001], humanoid robot JAST [F(1,23) = 5.54; p = 0.027], rotated industrial robot arm JAHIR 90° [F(1,9) = 6.77; p = 0.029], but not JAHIR [F(1,18) = 1.34; p = 0.26 n.s.] (cf. Fig. 5). In both direct comparisons human-JAST and human-JAHIR 90°, the interaction AGENT×CONGRUENCY was not significant (both p>0.54), showing that there was no difference in MI between the human agent and these robots. In contrast, the comparison human-JAHIR yielded a significant interaction AGENT×CONGRUENCY [F(1,18) = 7.11; p = 0.016], confirming that MI was not present for JAHIR. Since the industrial robot was the same in both presentations – mounted on the table in JAHIR and rotated in JAHIR 90° – this result strongly suggests that a human-like joint configuration (with respect to the observer) is a crucial factor for triggering MI.

Finally, to test for the presence of facilitation effects on one’s own movement during observation of congruent movements of a different person, which would manifest in a more accurate movement in comparison to baseline where no other person is present, we used a repeated measures ANOVA with factors AGENT PRESENCE (agent/baseline) and DIRECTION (vertical/horizontal) for data obtained while watching incongruent and congruent movements of a human agent and baseline data. The effect of AGENT PRESENCE could be shown only in the incongruent condition [F(1,24) = 5.6 p<0.026] with a higher variance in one’s own movement during observation of incongruent movements than during the baseline. However, no additional accuracy in case of congruent movement observation could be shown [F(1,24) = 0.37; p>0.54].

### Other measures of MI

**Figure 6:**
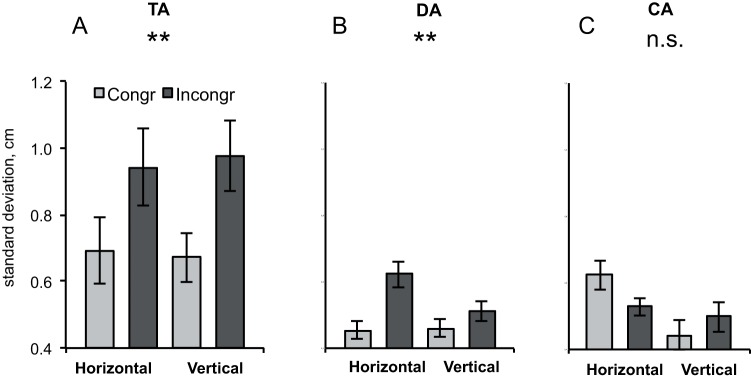
TA, DA and CA analyses for human agent observation. A: Actual plane tilt of movement (mean line fit) with respect to the horizontal and vertical directions (TA). B: Standard deviation of individual line fit from the mean line fit (actual plane of movement) (DA). C: Curvature of single movements with respect to a straight line (individual line fit) (CA). Error bars represent standard error of the mean. Stars denote significance (** p<0.01).

To further investigate whether the deviations from the movement plane (DA), tilt of the movement plane with respect to the coordinate system (TA) and the curvature of the movement (CA) are differentially influenced by observation of congruent and incongruent movements, we performed separate analyses (repeated measures ANOVA) of our data while observing a human with PLANE (horizontal/vertical) and CONGRUENCY (congruent/incongruent) as within-subject factors. This analysis revealed an effect of congruency for DA [F(1,32) = 27.7; p<0.001] (see Fig. 6a) and for TA [F(1,32) = 9.6; p<0.005] (see Fig. 6b) but not for CA [F(1,32) = 0.376; p<0.8] (see Fig. 6c). For DA there was an additional effect of direction [F(1,32) = 7.2; p<0.011] due to higher deviation in the horizontal plane than in the vertical plane and an interaction between direction and congruency [F(1,32) = 8.0; p<0.008] due to a stronger effect of incongruence in the horizontal than in vertical plane.

Correlation analysis (corrected p-level p = 0.004 for 12 tests) of the different factors contributing to MI has shown a significant positive correlation between SA and TA in four conditions (all n = 33 subjects): HC [r = 0.681, p<0.001]; HI [r = 0.786, p<0.001]; VC [r = 0.484, p<0.004]; and VI [r = 0.760, p<0.001]. SA and DA also correlated in all conditions (HC [r = 0.409, p = 0.018]; HI [r = 0.386, p = 0.026]; VC [r = 0.592, p<0.001]; and VI [r = 0.460, p = 0.007]), even though only VC was significant due to the Bonferroni correction. Similarly, SA and CA significantly correlated only in the vertical conditions: HC [r = 0.488, n = 33, p = 0.037]; HI [r = 0.120, n = 33, p = 0.5060]; VC [r = 0.769, n = 33, p<0.001]; and VI [r = 0.540, n = 33, p = 0.001].

Finally, a multiple regression analysis was used to test if the factors curvature, movement variability and plane tilt significantly predicted the SD of the movement with respect to the horizontal and vertical axis respectively during observation of congruent horizontal human movements. The results of the regression indicated that the three predictors explained more than 88% of the variance of the standard analysis in each condition with TA and DA contributing most.

## Discussion

The present study strongly suggests that MI depends on the configuration of the motor system of the observed agent, i.e., its joint configuration, rather than on the presence of human-like features such as a body with two hands and a head. The same industrial robot arm performing exactly the same movements induced MI when it had human-like motility, i.e. when it was presented in a joint configuration similar to the human arm (tilted by 90°, see Fig. 2d), but not when it was shown in the standard industrial configuration (see Fig. 2c). Although the kinematics of the end effector (the gripper) of the robot arm did not change relative to the observer in the two configurations, only the robot arm in the tilted, human-like joint configuration moved in a way which resembled a human arm movement.

In addition, the present result confirm our previous finding [13] that MI does not depend on the characteristic movement variability of human motion but can be elicited while observing a robotic arm moving with a stereotyped *quasi-biological* velocity profile. MI elicited by watching videos of human arm movements was consistent with previous studies [10], [13], [20], [22], [29], confirming that movement observation significantly interferes with ongoing executed movements, if the observed movements are qualitatively different from the movements produced.

We therefore suggest that MI is not due to the biological nature of the observed agent or its human-like appearance, but rather its human-like motility. Thus, the importance of good match between the motor systems of the actor and observer during action observation supports and is consistent with the simulation theory, which indicates that for action understanding the observed actor should have the same motor constraints as the observer [30]. This suggestion is also in line with previous studies showing that in infants simulation cannot take place when the observed action cannot be transformed to the own body, as in case of geometrical shapes [31], mechanical devices [30], or claws [32], [33]. On the contrary, visual identification of an agent with a human-like body structure, like in case of humanoid robots [31], might enable children to simulate the observed actions and map them isomorphically to our bodies. Thus, it is conceivable that in the original study by Kilner et al. [10] MI was absent during observation of robotic arm movement not only because of constant movement velocity, but also because the robot's artificial joint configuration did not allow observers to translate movements to the human body. In contrast to Albert et al. [18], who suggested that human shape is a crucial factor in triggering MI, and Chaminade & Cheng [34], who claimed that MI can be triggered only when the whole body is visible, our study shows that a human-like joint configuration combined with smooth movements is sufficient to elicit MI. Combination of our results regarding agent shape, motility and movement kinematics with previous studies investigating the effect of movement velocity profile [11], [40] and agent shape [41] indicates that both human-like joint configuration and at least a quasi-biological movement are required for triggering MI and that even a high degree of one cannot compensate for the absence of the other. In other words, even a very high degree of human likeness of an agent is not sufficient to trigger MI, if its movements are not smooth enough [41].

A different possibility to explain our results has been suggested by Shen et al [14] who claimed that the in human–humanoid interaction perception of an agent as a “social entity”, for example, due to the observer’s beliefs, is critical for eliciting MI rather than any individual appearance or motion feature. While this may hold for our experiment with the humanoid robot, it is difficult to account with this theory for all our findings. In particular, perception of being a “social entity” can hardly explain why the video recording of the detached industrial robot arm elicited MI when it was turned 90 deg, but not in the original version (Fig. 2C and 2D). We therefore argue that it was the human-like motility of the arm in the rotated version, which increased MI, but not a change in the observer’s belief about the agent being a social entity or not.

The main function attributed to motor resonance is action understanding, since mirroring the actions of others might help to understand what another person is doing [8] and why he/she is doing it [7]. Thus, simulating another person’s actions might allow humans to make predictions about the mental states of others based on the mental states and behaviors that they experience themselves while mimicking others [23], [24]. Research on visuomotor priming has shown that responses to human hand movement stimuli (e.g. a video image of a hand opening) are faster and more accurate when they involve execution of the same movement (e.g. hand opening) than when they involve execution of an alternative movement (e.g. hand closing) [25], [26]. Similarly, if the subjects are instructed to perform a finger tapping in response to a visual signal depicting finger tapping (compatible) or lifting (incompatible), the reaction time to initiate the prepared finger movement significantly slows down when the stimulus is incompatible [2]. Individuals automatically mimic many different aspects of their interaction partners, including speech patterns, facial expressions, emotions, moods, postures and gestures [27] and the higher degree of movement synchronization (chameleon effect) between interaction partners is generally regarded to be a sign of higher degree of mutual rapport, involvement and togetherness [27], [28].

All these findings indicate that MI, which can be seen as the consequence of the tendency to mimic other people’s actions, can be used as an indicator of the easiness and intuitiveness of interaction with other agents. Capa et al. [19] have shown that MI is likely to arise from activity in the mirror neurons, which are supposed to be the neural basis for motor simulation [8]. In their study, MI increased in observers who had previous extensive practice with the observed movement in comparison to naive observers, indicating that visuo-motor experience facilitated motor resonance with the observed movement. The hypothesis that observation of an industrial robot arm may trigger the same type of implicit perceptual processes as a human agent is in line with recent studies providing evidence that observing actions produced by robot arms [35], [36] and humanoid robots [37], [38], [39] leads to comparable activations in the MNS as observation of human actions.

Most previous studies used a measure for MI that is dependent on a space-fixed coordinate system, i.e., the deviation of subjects’ movement trajectory from the instructed movement plane (SA). Since such an analysis depends on accurate alignment of movement directions between the subject and the observed agent, we tested which components of the movement contribute to MI. The analysis of the three contributing factors indicated that SA correlated with the movement curvature (CA), tilt (or shift) of the overall movement plane in respect to the vertical or horizontal plane (TA) and deviations of individual movements from the overall movement plane (DA). As expected, the regression analysis showed that the combination of the three factors DA, CA and TA explained approx. 90% of the movement trajectory deviations from the instructed movement plane. However, the curvature of the individual movements (CA) contributed only negligibly to the overall effect. The DA analysis revealed a significantly higher SD in the horizontal than in the vertical plane (see Fig. 6b), which might be due to a difference in the biomechanical properties of forearm movements in horizontal and vertical planes or due to the fact that the deviations during horizontal movement might have been facilitated by gravity. Since the overall tilt from the instructed movement direction (TA) plays such an important role in MI, future investigations need to assure careful calibration of the spatial coordinates of both the movements of the observed agent and of the test subjects.

### Conclusions

The results of the present experiments show that MI, which is explained by the motor resonance hypothesis, is not specific to human–human interactions but can also be observed in interactions with inanimate agents. Together with previous studies, our study suggests that the combination of a human-like joint configuration and biological motion of the observed agent, i.e., its motility, rather than its human-like appearance may be the most important factor for action understanding and perhaps even for joint interaction.
